# Refractive Index and Temperature Sensing Performance of Microfiber Modified by UV Glue Distributed Nanoparticles

**DOI:** 10.3390/polym14122425

**Published:** 2022-06-15

**Authors:** Hongtao Dang, Yan Zhang, Yukun Qiao, Jin Li

**Affiliations:** 1 Shaanxi Engineering Research Center of Controllable Neutron Source, School of Electronic Information, Xijing University, Xi’an 710123, China; skydht@163.com (H.D.); zy353615@163.com (Y.Z.); qiaoyukun6530@163.com (Y.Q.); 2 College of Information Science and Engineering, Northeastern University, Shenyang 110819, China; 3 Hebei Key Laboratory of Micro-Nano Precision Optical Sensing and Measurement Technology, Qinhuangdao 066004, China

**Keywords:** silicon nanoparticles, surface plasmon resonance, microfiber sensor, refractive index sensing, temperature sensing

## Abstract

Dielectric materials with high refractive index have been widely studied to develop novel photonic devices for modulating optical signals. In this paper, the microfibers were modified by silicon nanoparticles (NPs) and silver NPs mixed in UV glue with ultra-low refractive index, respectively, whose corresponding optical and sensing properties have been studied and compared. The influence from either the morphological parameters of microfiber or the concentration of NPs on the refractive index sensing performance of microfiber has been investigated. The refractive index sensitivities for the microfiber tapers elaborated with silver NPs and silicon NPs were experimentally demonstrated to be 1382.3 nm/RIU and 1769.7 nm/RIU, respectively. Furthermore, the proposed microfiber was encapsulated in one cut of capillary to develop a miniature temperature probe, whose sensitivity was determined as 2.08 nm/°C, ranging from 28 °C to 43 °C.

## 1. Introduction

Gold and silver nanoparticles (NPs) can confine the light field in the sub-wavelength scale to overcome the optical diffraction limit due to the localized surface plasma resonance (LSPR) excited at their surface [1]. LSPR based optics modulation has been widely involved in astrophysics, life science, optics, photonics, materials and other fields [2,3]. Metal or metal oxide NPs can be doped in polymer gel [4] and elaborated on the surface or inside of the optical fibers to develop compact sensing probes [5,6]. A highly sensitive performance has been experimentally demonstrated in many works reported in recent years [7,8,9,10], in which noble metal or metal oxide nano-films or NPs were elaborated on the different fiber structures (microfiber taper, D-shaped, U-shaped interface) to excite long range surface plasmon resonance (LRSPR) or LSPR effect [11]. These fiber sensors have been used for detection of analytes such as hormones, contaminants, etc. [12]. In addition to traditional noble metal NPs, other metal oxides (SnO_2_, WO_3_, etc.) also exhibit excellent sensing performance [13,14]. Various NPs have been doped into silica or polymer optical fibers for determination of bio-molecular or chemical composition [15,16,17], where internal doping and surface coating have become the most typical modification methods. Compared with crystal or dielectric materials, the scattering loss of metal particles is extremely serious. During the LSRP excitation process, the transition of free electrons among different energy levels will increase the surrounding temperature, resulting in the Joule heating effect [18,19]. Although it has been proven to be a merit in photo-thermal cancer therapy, photo-thermal imaging and photo-thermal bio-sensing [20], the local heating is extremely unfavorable for photonics devices and sensors with high requirements for environmental stability, such as in vivo detection [21], where the local heater will deform or even erase the nanostructure, thereby affecting the optical modulation performance of the photonic device [22]. More issues can be found in other works, for example, it may change the refractive index of the sensing material or structure due to the thermo-optical effect [23], and it can volatilize the liquid and cause thermal damage to in vivo samples [24]. In addition, LSPR devices also suffer due to poor compatibility with the semiconductor’s oxide. To overcome the optical loss and self-heating problem, dielectric materials with high refractive index have been introduced, including silicon, germanium, gallium phosphide, etc. The magneto-optics and direction-scattering characteristics for all-dielectric NPs or structures have been verified [25,26,27]. Near field optical manipulation has been demonstrated based on dielectric NPs with high refractive index, which has proved a promising prospect in solving the scattering loss and self-heating problem in photonics devices.

Mie scattering mainly depends on dielectric constant and size for lossless, non-magnetic NPs. The maximum scattering efficiency of subwavelength NPs is only related to the resonance frequency and is irrelevant to the material [28]. Therefore, the LSPR-like effect, similar to that of metal NPs, can also be realized for dielectric NPs with a high refractive index. The scattering efficiency will be significantly enhanced due to the higher refractive index [29,30]. It has been revealed that all-dielectric nanostructures have similar characteristics to plasmonic devices, including enhanced scattering, high-frequency magnetic fields, and negative refractive index [31]. By precisely designing the structures and optimizing their parameters, all-dielectric nanostructures display an even better performance [32,33]. Furthermore, dielectric NPs (or structures) can play a similar role to plasmonic devices, being self-assembled (or processed) to act as photonic crystals and meta-surfaces on 2D substrate or optical fibers [34,35].

In recent years, most of the related research on the nonlinear optical properties and sensing applications of dielectric nanostructures have been focused on the meta-surface structure on planar substrates. The controllable modification and sensing application of all dielectric nanoparticles with high refractive index are rarely reported. In this work, silicon or silver NPs have been elaborated on the surface of microfibers and their corresponding refractive index sensing performance experimentally characterized. The influence of NPs’ concentration on the sensing performance has been verified to optimize the sensitivity. Based on the refractive sensing mechanism, a temperature probe was experimentally demonstrated as the Si NPs’ elaborated microfiber probe.

## 2. Materials and Methods

**Figure 1:**
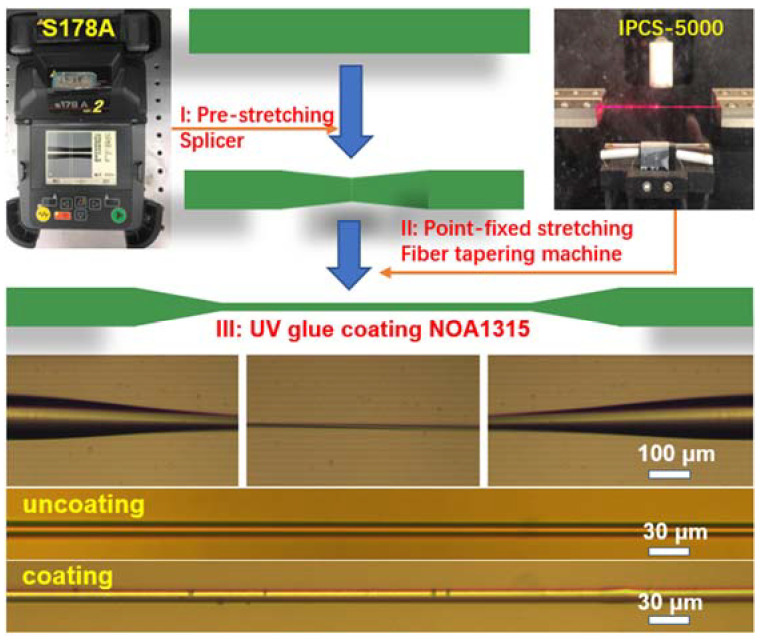
Schematic diagram of fabrication process of NPs-coated microfiber using two-steps point-fixed stretching method.

The microfiber was fabricated by a two-steps point-fixed heating-drawing technique, from the common single-mode fiber (SMF-28, Corning Inc., Corning, NY, USA) with refractive index of 1.4682 and 1.4628 for the corresponding core and cladding, respectively. The preparation process of the microfiber taper is divided into two main steps, as shown in Figure 1: pre-stretching by a fiber splicer, and point-fixed stretching by a fiber tapering machine.

The single-mode fiber taper is made via a manual mode using a fiber splicing machine (Furukawa, S178A, Furukawa Electric Co., Ltd., Tokyo, Japan), where the discharging time and moving process of the fiber holders can be flexibly adjusted. In detail, the ordinary single-mode optical fiber was fixed in the fiber splicing machine. After selecting the corresponding optical fiber matching mode, the working mode was changed to manual operation. The pulse value of two fixed motors Z_L and Z_R was set as ~3400. After repeating the arc discharges twice, the bi-conical microfiber with a waist diameter of ~40 μm could be obtained; the same single-mode fiber taper was scan-heated and further stretched by a fiber stretching machine (Idealphotonics, IPCS-5000, Idealphotonics Inc., Vancouver, BC, Canada) to obtain microfiber with a diameter of less than 5 μm and a length of 5–8 mm.

A hydrogen generator was used to provide the hydrogen-oxygen flame with temperature up to 1500 °C. During the heating-stretching process, the torch scans in a fixed region, where the desired fiber length and scanning range can be set via the operating board. The target parameters (fiber length) are easily obtained by the equal volume calculation. Meanwhile, the scanning range should be limited to less than the length of the thin waist region to maintain the uniform diameter of the long-tapered microfiber. In order to effectively reduce the optical loss and improve the binding efficiency of the light field, UV glue with ultra-low refractive index (NOA1315, refractive index is 1.315, Norland Products Inc., Jamesburg, NJ, USA) was introduced to disperse the silver or silicon NPs and uniformly elaborate them on the outer surface of the microfiber region. NOA1315 has good fluidity due to its ultra-low viscosity of only 15 cps. The NPs were added and the mixture was dispersed by magnetic stirring for 10 min to distribute the NPs uniformly. The UV glue-NPs mixture was injected into a syringe and dripped onto the surface of the microfibers, whose surface morphology can be compared according to the microscope pictures in Figure 1.

**Figure 2:**
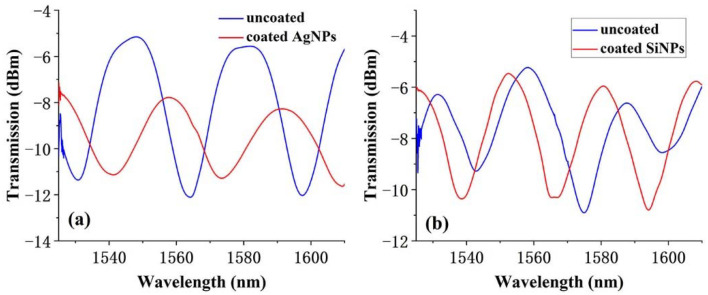
Spectra comparison of a clean microfiber taper with an elaborated microfiber taper by (**a**) silver and (**b**) silicon NPs.

The transmission spectra of clean microfiber tapers were compared with those of microfibers elaborated by NPs, as shown in Figure 2a,b, respectively.

The significant influence on the spectra is revealed, due to the introduction of the UV/silver or UV/silicon NPs composite film. The interference spectrum still has a good waveform in the later experiment. To accurately control the sensing length, too long or too thin microfiber was excluded due to poor fabrication repeatability. In this work, the microfiber length was strictly controlled at ~6 mm to obtain a stable spectrum with the relative-fixed free range (FSR) of ~40 nm. During the first step, the manual mode of the fiber splicer was used to fabricate a short taper with waist diameter of ~50 μm, as the inserted image shows. This short taper was further stretched using the flame scanning method by a multifunctional fiber drawing machine. Then, the flame torch was, respectively, fixed on point 1 and 2 to obtain microfiber double-tapers with thinner diameter of ~10 μm.

In Mie’s theory, the extinction spectra *Q_ext_* of the spherical NPs can be presented [36] as

(1) $$Q_{ext}=\frac{18\pi \varepsilon_{m}^{3/2}V}{\lambda }\cdot \frac{\varepsilon_{2}\left(\lambda \right)}{{\left[\varepsilon_{1}\left(\lambda \right)+2\varepsilon_{m}\right]}^{2}+\varepsilon_{2}^{2}\left(\lambda \right)}$$

Here, *ε_m_*, is the dielectric constant of the surrounding environment; *ε*_1_ and *ε*_2_ refer to the real and imaginary part of the complex dielectric constant, respectively; *λ* is the working wavelength; and *V* is the volume of spherical NPs. In periodic metal nanostructures, the excitation spectra depend on the structure size [37]. However, in our proposed microfiber sensor, it mainly relies on the multimode interference (MMI) effect to detect the change in external refractive index [38], where the wavelength shift of the MMI spectrum (Δ*λ*) depends on the change of the equivalent refractive index (*n_eff_*) of the surrounding medium environment, which can be expressed as [39]

(2) $$\Delta \lambda =p\frac{n_{eff}W_{eff}^{2}}{L}$$

where *p*-th is the image of the input field; *L* is the MMI length along the microfiber; *W_eff_* is the effective optical diameter of microfiber, and depends on the physical diameter *W*, the refractive index of core *n_r_* and the cladding *n_c_* of the microfiber. This value can be calculated by [40]

(3) $$W_{eff}=W+\frac{1}{2}\left(\frac{\lambda_{0}}{\pi }\right){\left(n_{r}^{2}-n_{c}^{2}\right)}^{-1/2}\left[{\left(\frac{n_{c}}{n_{r}}\right)}^{2}+1\right]$$

Here, *λ*_0_ is the working wavelength in free-space. Because the diameter of the microfiber is very small, its core and cladding have been integrated during the fiber stretching process. The evanescent field will propagate along the microfiber, and the refractive index of the whole microfiber will be usually equivalent to the same value. In this case, it assumes that *W_eff_* ≈ *W* [38]. The second term of Equation (3) will become significant with the decrease in W; therefore, the sensitivity of the MMI based microfiber can be improved by reducing the diameter of the microfiber and extending the sensing region.

## 3. Results and Discussion

### 3.1. Refractive Index Sensing Performance Comparison

**Figure 3:**
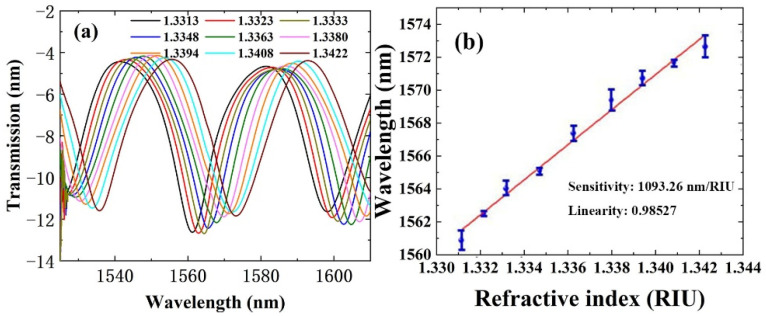
Refractive index sensing performance of a UV-glue-coated-microfiber. (**a**) Spectra change as a function of refractive index change from 1.3313 to 1.3422 and (**b**) corresponding sensing characteristics curve.

The refractive index sensing performance of a UV-glue-coated-microfiber long-taper with the length of ~6 mm has first been demonstrated. In Figure 3a, the transmission spectra red-shifted when the refractive index changed from 1.3313 to 1.3422. The corresponding sensitivity was determined to be 1093.26 nm/RIU for the linear fitting results, shown in Figure 3b. It should be noted that the introduction of the UV glue layer with low refractive index resulted in an interference spectrum with high quality.

**Figure 4:**
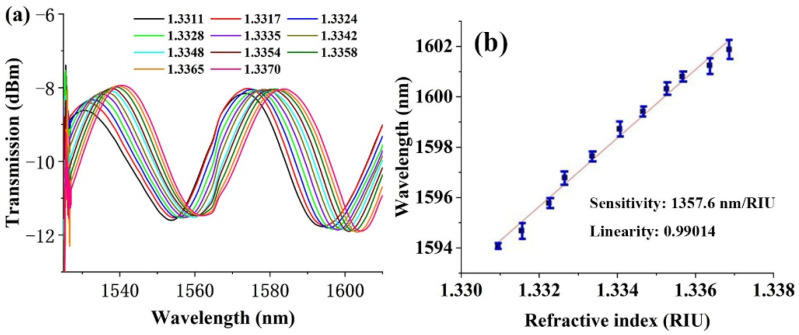
(**a**) Interference spectra and (**b**) responding curve as a function of refractive index for microfiber taper elaborated by silver NPs with concentration of 0.1 mol/mL.

Furthermore, the UV-glue-coated-microfiber can sense the refractive index change for its environment. The influence of NPs’ concentration on the optical and sensing performance of the function film coated microfiber should be studied. Too low concentration will support a weaker LSPR effect or LSPR-like effect for either silver or silicon NPs, which will also exert little impact on the sensing performance. By contrary, too high concentration will introduce a serious loss signal due to the light scattering on the microfiber surface. The concentration range was finally chosen in the range of 0.01 mol/L–0.5 mol/L in this experiment. The output spectrum of the silver NPs’ (0.01 mol/L) elaborated microfiber is shown in Figure 4a. The sensitivity is determined to be 1382.3 nm/RIU after linear fitting (Figure 4b) of the experimental data for the wavelength shift. During the experiment, different refractive index environments are provided by the slowly cooling distilled water from 40 °C to room temperature, so as to reduce the influence of experimental environment fluctuation on the transmission spectrum of microfiber.

**Figure 5:**
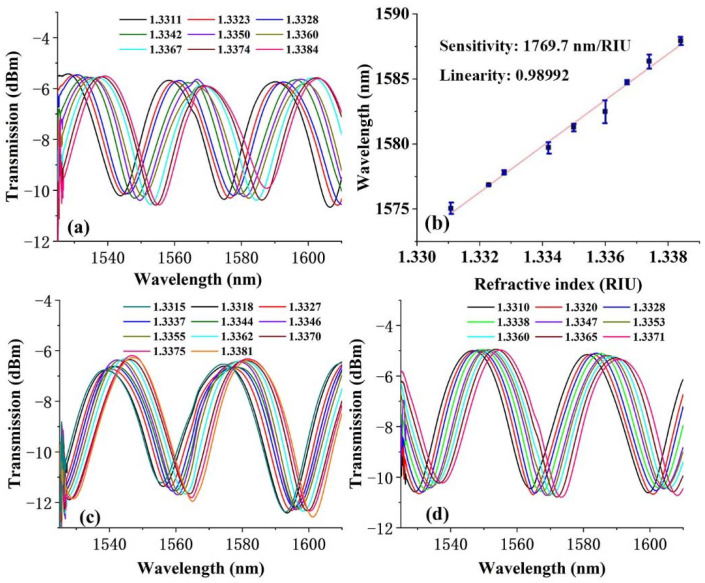
(**a**) Interference spectra as a function of refractive index and (**b**) corresponding fitting curve for microfiber taper elaborated by silicon NPs with a concentration of 0.01 mol/mL. (**c**,**d**) Interference spectra for other two probes with similar morphological parameters and coating layers.

The refractive index values were measured by an Abe refractometer (WYA-2S, Shanghai Yidian physical optical instrument Co., Ltd., Shanghai, China); meanwhile, the corresponding spectra were recorded by a spectrum analyzer. Further experimental results show that, with the increase of concentration, as compared in Table 1, this sensitivity cannot be further enhanced. At low concentrations (0.01 mol/L, 0.05 mol/L, 0.1 mol/L), the sensitivity is ~1360 nm/RIU with a good linearity of R2 = ~0.99. When the concentration is 0.5 mol/L, the sensitivity has the highest value of up to 1842.3 nm/RIU, R2 = 0.99167. Figure 5a shows the output spectrum of the microfiber elaborated by silicon NPs with a concentration of 0.01 mol/L. The experimental fitting line reveals a refractive index sensitivity of 1769.7 nm/RIU in Figure 5b. Further experimental results indicate that, as the concentration increases, the sensitivity was reduced significantly.

It should be noticed that the preparing process of the proposed microfiber RI sensor is controllable either for the fabrication of microfiber (the parameters of fiber splicer and fiber tapering machine will be fixed), or for the UV-glue coating process (the mass of NPs and volume of UV-glue can be determined). The spectra response of two more probes (with a diameter of ~10 μm, length of ~6 mm and similar silicon NPs concentration of 0.01 mol/mL) are included in Figure 5c,d. Their spectral waveforms show little difference, where the differentiated positions of the peaks and dips are affected by the scale difference (diameter and length) of the microfiber, which is less than one quarter of the optical wavelength. The wave spectra and corresponding response to refractive index change are similar to each other. More than five rounds of experiments to prepare microfiber sensors of the same size and similar coating film, the RI sensitivity difference was limited to less than 100 nm/RIU. For the highest concentration of 0.5 mol/L, the corresponding sensitivity was 1321.8 nm/RIU. The decreasing sensitivity may have contributed to the evanescent field scattering by the high dose of silicon NPs. However, in general, the introduction of the silicon NPs significantly improved the sensitivity. Each sensor probe will be calibrated separately before it is used. During the detection process for refractive index, the specific value of refractive index is calculated by the relative wavelength shift of special peaks or dips. Therefore, the little differences in either spectrum or sensitivity for different probes will not exert a significant effect on their refractive index sensing performance.

Compared with the previous experimental data, it can be concluded that the refractive index sensitivity of a clean microfiber without coating layer is 1093.3 nm/RIU. The comparison of the sensing performance of the microfibers elaborated by silver and silicon NPs with different concentrations is shown in Table 1.

**Figure 6:**
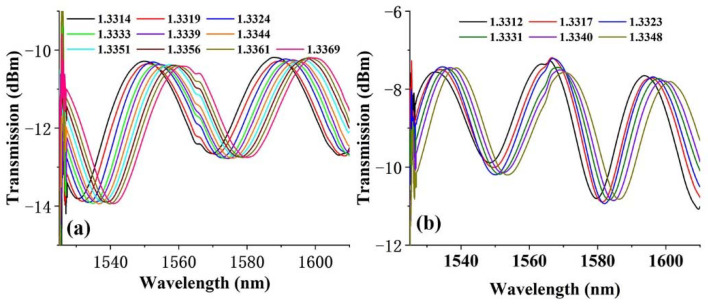
Interference spectra as a function of refractive index of the microfiber elaborated with (**a**) silver and (**b**) silicon NPs with same concentration of 0.5 mol/L.

The spectra of microfiber elaborated by silver and silicon NPs with the same concentration of 0.5 mol/L can be seen in Figure 6. Although the microfiber is destroyed during the measurement process, and the silicon NP modified microfiber had only six spectra corresponding to different refractive indexes, the monotonic and significant refractive index response curve can be obtained, with corresponding sensitivity of 1321.8 nm/RIU.

It was found that the microfiber elaborated by silver NPs has the highest sensitivity of 1842.3 nm/RIU; for silicon NPs, the highest sensitivity is up to 1769.7 nm/RIU. It is revealed that, after being elaborated by the NP layer, the refractive index sensitivity of microfiber was significantly improved. NPs have a significant effect on optimizing the sensing performance. Furthermore, the dielectric NPs have a similar LSPR effect, similar to that of metal NPs. The refractive index sensing curve has also been verified with a high linearity coefficient R2. The sensitivity of the proposed microfiber sensors has been compared with those of other metal NPs-based fiber sensors in Table 2.

The sensitivity can be theoretically increased to 104 levels by the compositing of other function materials with the metal NPs. The experimentally demonstrated sensitivity for the Ag NPs and Au NPs ranges from 349.1–8600 nm/RIU to 900–5140 nm/RIU, respectively, referring to the earlier review (Ref. [2]). The sensitivity in this work is comparable with that reported in the references. In this work, the silicon NPs were elaborated on the microfiber to experimentally demonstrate its possible LSPR-like effect, similar to that of metal NPs. This sensitivity can be further improved by reducing the diameter of the microfiber or introducing other function materials.

### 3.2. Temperature Sensing Properties Analysis

**Figure 7:**
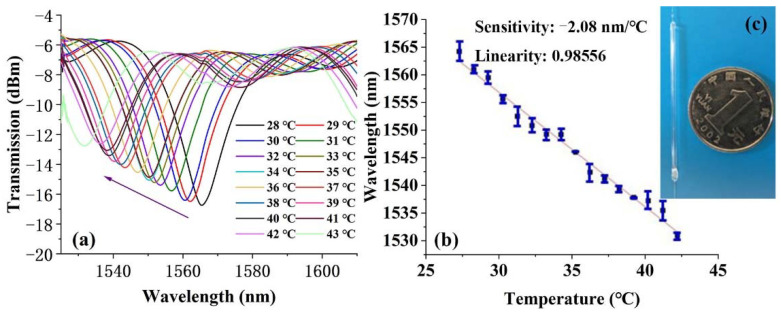
(**a**) Interference spectra as a function of environment temperature and (**b**) temperature sensing characteristic curve for microfiber taper elaborated by silicon NPs during 28 °C–43 °C. (**c**) Picture of microfiber packaged in glycerin by a capillary.

A high-sensitivity temperature probe was proposed by encapsulating the silicon-NPs-elaborated-microfiber sensor with high refractive index sensitivity in one cut of silica capillary filled with glycerin. Both ends of the capillary were sealed by photosensitive glue. The refractive index of glycerin is inversely proportional to the temperature, which supplies the refractive index changing environment. Although the refractive index of glycerol is greater than that of silica micro fiber, a certain amount of optical signal is still limited near the interface of the low refractive index UV coating layer and silica microfiber in the form of evanescent field. The spectra as a function of temperature curve are shown in Figure 7 during 28 °C–43 °C.

The photo of the microfiber encapsulated in the silica capillary is illustrated in Figure 7c. Its actual size can be determined by comparing with the 1 Yuan RMB coin. In the temperature range of 28 °C–43 °C, the wavelength blue-shifted by 2.08 nm for every 1 °C of temperature decrease, resulting in a temperature sensitivity of 2.08 nm/°C. High sensitivity also means a narrow working range of between 1520 nm–1610 nm. Although the sensitivity of the proposed temperature probe is verified in a limited range (28 °C–43 °C), normally it may work in a larger temperature range by demodulating the phase change of the spectral wavelength. For this temperature probe, the temperature sensing performance is mainly limited by the thermal stability of glycerol. Relevant studies show that the water in glycerol aqueous solution will evaporate rapidly when the temperature is higher than 120 °C [41], and the oxidation temperature of glycerol is about 150 °C. Therefore, the probe may work normally at a temperature less than 100 °C. Therefore, it will be suitable for high-precision monitoring of temperature fluctuations in special areas desiring a high sensitivity, rather than a wide working range.

## 4. Conclusions

In this paper, silver NPs and silicon NPs were uniformly dispersed in low-refractive-index UV glue and coated on the surface of a microfiber to prepare refractive index probes with high sensitivity. The corresponding refractive index sensitivities of 1842.3 nm/RIU and 1769.7 nm/RIU have been experimentally obtained, respectively, for silver NPs’ and silicon NPs’ elaborated microfiber with a diameter of ~10 μm and length of ~6 mm. The silicon NPs’ modified microfiber with high refractive index sensitivity has also been used for determining environmental temperature with a sensitivity of 2.08 nm/°C. The sensing probe is easily obtained from ordinary single-mode optical fiber, and can be easily connected into the conventional fiber network for exploring high-performance sensors and photonic devices.

## Figures and Tables

**Table 1:** Sensing performance comparison for 6 mm length of microfiber elaborated by silver and silicon NPs with different concentrations.

Materials	NPs Concentration	Sensitivity	Linearity
Silver	0.01 mol/L	1382.3 nm/RIU	0.99291
	0.05 mol/L	1364.8 nm/RIU	0.98768
	0.1 mol/L	1357.6 nm/RIU	0.99014
	0.5 mol/L	1842.3 nm/RIU	0.99167
Silicon	0.01 mol/L	1769.7 nm/RIU	0.98992
	0.05 mol/L	1471.8 nm/RIU	0.98968
	0.1 mol/L	1468.1 nm/RIU	0.99534
	0.5 mol/L	1321.8 nm/RIU	0.99252

**Table 2:** Comparison of sensitivity for reported works and proposed sensor.

Sensitive Materials	Sensitivity	Refs.
Bi-layered Au NPs	49.63 a.u./RIU	[1]
Ag NPs based works	349.1–8600 nm/RIU	[2]
Au NPs based works	900–5140 nm/RIU	[2]
Au film (40 nm)	~2459–20,863 nm/RIU #	[4]
Au NPs + ZnO NPs	~6 nm/μM	[5]
Au film + TiO_2_ layer	30,000 nm/RIU #	[7]
Ag NPs	1842.3 nm/RIU	This work
Si NPs	1769.7 nm/RIU	

# Simulation results.

## Data Availability

Not applicable.
